# CircMMD_007 promotes oncogenic effects in the progression of lung adenocarcinoma through microRNA-197-3p/protein tyrosine phosphatase non-receptor type 9 axis

**DOI:** 10.1080/21655979.2022.2037956

**Published:** 2022-02-14

**Authors:** Lihuan Zhu, Tianxing Guo, Wenshu Chen, Zhaoxian Lin, Mingfan Ye, Xiaojie Pan

**Affiliations:** Department of Thoracic Surgery, Fujian Provincial Hospital, Shengli Clinical Medical College of Fujian Medical University, Fuzhou, Fujian Province, China

**Keywords:** circMMD_007, lung adenocarcinoma, miR-197-3p, PTPN9, oncogenic

## Abstract

Circular RNAs play important roles in cancer biology. In this research, we explored the underlying function and mechanism of cirMMD_007 in lung adenocarcinoma (LC). Clinical lung adenocarcinoma samples were obtained from surgery. Bioinformatic databases were used to predict miRNAs that can potentially target circRNAs and miRNA target genes. hsa_circMMD_007, miR-197-3p, and PTPN9 mRNA expressions were investigated by qRT-PCR. Protein expressions were examined using Western blot. The proliferation abilities were assessed by Cell Counting Kit-8 assays. Wound healing cell migration assay was applied to evaluate cell migration ability. Luciferase reporter assay and rescue experiments were then performed to elucidate the underlying mechanism. We found that the expression of circMMD_007 was abnormally increased in LC. The expression of circMMD_007 was higher in advanced stages. Knockout of circMMD_007 hindered the tumorigenesis of LC in vivo and in vitro. circMMD_007 could negatively regulate the expression of miR-197-3p. PTPN9 behaved to be a molecular target of miR-197-3p. In summary, this research demonstrated that circular RNA circMMD_007 could promote the oncogenic effects in the progression of LC through miR-197-3p/PTPN9 axis.

## Graphical Abstract

**Figure:**
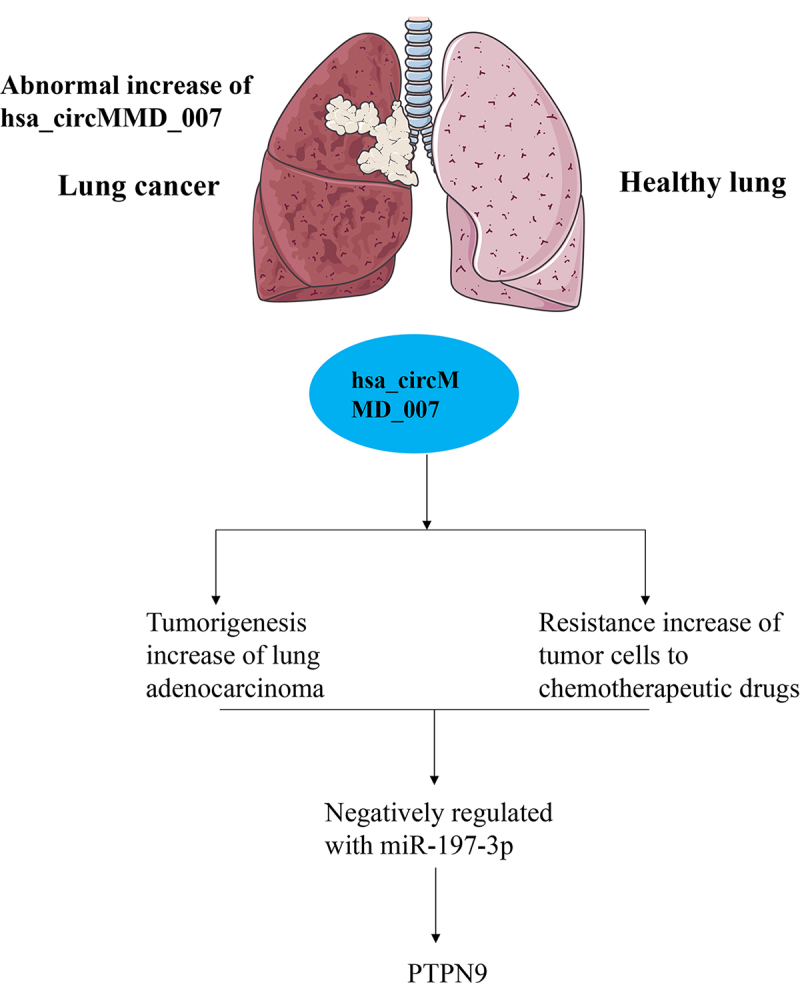

## 1. Introduction

Globally, lung cancer is found to be a widespread disease [1]. Lung adenocarcinoma (LC) represents almost 40% of all kinds of lung cancers. It is one of the utmost commonly occurring subtypes of cancer especially in the nonsmokers [2]. LC rarely occurs under the age of 20, the average age of its diagnosis is considered to be 71 years [3]. The five-year survival rate in patients suffering from LC is still dismal, despite recent breakthroughs in the field. It normally originates from the mucosal glands and usually ensues in the periphery of the lung [4]. The appearance of physical signs and the symptoms depends on the stage of lung cancer. Patients are usually asymptomatic in the initial stages of the disease and can present with common symptoms of cough, hemoptysis, and unplanned weight reduction in the advanced stage of the disease [1]. For patients suffering from Stage 1–Stage 3A LC, surgical treatment is required. Chemotherapy is administered for cases of LCs, which are either untreatable or diagnosed at a later stage [5]. Patients who test positive for ALK mutation and EGFR mutation can be treated using ALK inhibitors and tyrosine kinase inhibitors based first-line chemotherapy, respectively [6]. A platinum-based doublet first-line chemotherapy is advised with bevacizumab as a possible third agent, in case a patient exhibits tumor, which is ALK and EGFR negative [5]. The survival rate of patients suffering from LC is far from promising, regardless of the substantial advancements in chemotherapy and molecular targeted therapy. Thus, developing new therapeutic targets is crucial.

In recent years, due to the emerging sequencing and bioinformatics technologies, a variety of circRNAs have been discovered to regulate several biological processes by acting as critical modulators. Unlike linearly structured RNAs, circular RNAs have covalently bonded ends and have better stability and expression [7]. The existence of circular RNAs, which can be released in saliva and blood, has been confirmed, and they are mainly contained in the cytoplasm [8]. These features of circular RNAs will mark them as active diagnostics and prognostic indicators for several diseases, especially cancer [9]. Exploring the potential mechanism of active circular RNA will benefit research of LC.

In the present study, we aim to investigate if circular RNA circMMD_007 could promote the oncogenic effects in the progression of LC through miR-197-3p/protein tyrosine phosphatase non-receptor type 9 (PTPN9) axis. This study might provide more novel insight for the prevention and treatment of LC through regulating circMMD_007/miR-197-3p/PTPN9 axis

## 2 Methods

### 2.1 Clinical specimens and cell lines

The research was approved by the institutional research ethics committee of Fujian Provincial Hospital. The study consisted of 100 colorectal cancer diagnosed patients. All patients offered their written consent for participation. Samples of malignant cells as well as the surrounding healthy tissue were collected during surgery. Samples were then frozen immediately and transferred to the refrigerator for storage. Cell lines of human LC and normal cell line were obtained from ATCC. We cultured the cells in RPMI 1640 medium, 100 U/ml penicillin, 100 ug/ml streptomycin, and 10% fetal serum were added to the culture. Culturing of the cells was done at 37°C and humidity of 5% CO_2_ in an incubator.

### 2.2 Bioinformatics databases

CircInteractome [10] and Starbase [11] were used to predict the miRNAs, which can potentially target the circRNAs. miRDB [12], DIANA [13] database, and TargetScan [14] were used for predicting miRNA targets.

### 2.3 qRT-PCR

TRIzol was used to purify the total RNA from tumor tissues and cells. SuperScript First Stand Synthesis System was used to synthesize cDNA. Triplication of the qRT-PCR assay was done with the use of MyIQ Real-Time PCR Detection System. 2^−ΔΔCt^ method was used to calculate mRNA expression.

### 2.4 Western blot

One percent PMSF and RIPA lysine buffers were utilized to restrict the cellular proteins, which were then subjected to interaction with SDS-PAGE test buffer. The proteins were relocated to a layer of polyvinylide difluoride and these layers were permitted to grow overnight following 1 h of incubation at room temperature. Thereafter, the ECL chemiluminescence kit was applied, the proteins were fried with secondary antibodies for a duration of 1 h. GeneGnome 5 is utilized to assess the bands.

### 2.5 Transwell assay

We evaluated cell migration through the use of Oris Cell Migration Kit. Cell samples found in the 6-well plates developed to full confluence. A small section was disrupted by scratching the monolayer with a 200-μl plastic pipette tip. Samples were subjected to washing twice with a phosphate buffer saline and then transferred to a complete medium, which consists of various API levels. After 48 h wound closure was checked. Simultaneously images were taken with a phase-contrast microscope having a magnification of 100x. The cells were later washed using PBS.

### 2.6 CCK-8 assay

Cell count-kit-8 was used and the stable strains were digested using 1 ml of 0.25% trypsin, which then was transferred to a 15 ml centrifugal tube. It was centrifuged for 5 min at 1500 rpm. The resulting cells were seeded in six-well plates. Following 48 hours, the viability was assessed using CCK8. The 96-well plate then had addition of 10 μL of 5 mg/ml CCK8 solution, then it was subjected to incubation for a period of 2 hours in the dark. The absorbance level of individual well was assessed in the microplate at 450 nm.

### 2.7 Generation of lentiviruses and stable transfection

Purchase of miR-197-3p inhibitor and its parallel negative control was done by RiboBio. PolyJet Reagent was used for transfection of vectors and package of plasmids. Forty-eight hours later, the cancer cells were spin-infected on 12-well plates. Afterward lentiviral supernatants were centrifuged for 30 minutes. Harvesting of stable cell lines and cells was done using puromycin (3 ng/mL). Lipofectamine 2000 was used to conduct transient transfections.

### 2.8 RNA Immunoprecipitation (RIP)

Qiagen PCR Purification kit was used to cross-link the cells. Monolayer cells were scraped by a soft lysis buffer. After which the nuclei pellets were lysed with SDS lysine buffer. The protein was diluted to 1:10 using a dilution buffer then precleared using protein magnetic beads. Immunoprecipitation of the samples was done using antibodies. Following which the rescue of antibody chromatin complexes was achieved. Buffers were used to wash the beads. The eluted samples were de-crosslinked. The following day, the proteinase k solution was mixed with the protein complexes. It was then incubated for an hour. Following the manufacturers instructions, dual-luciferase reporter gene assay kits were used. The cells were co-transfected with circular RNAs. Two days later, RNA-binding protein immunoprecipitation was conducted to identify DNA targets of DNA-binding proteins with the help of a Magna RIP kit. The concentration of microRNA-197-3p was amplified following the purification of RNA complexes. The RNA-binding Protein Immunoprecipitation assay known as RIP assay for Argonaute RISC Catalytic Component Protein was conducted using an Ago2 antibody. Following the purification, the concentration of circMMD_007 and microRNA-197-3p was determined.

### 2.9 Dual-luciferase reporter assay

After transfection, signals were identified with a dual-luciferase reporter assay kit following the protocols provided by the manufacturer. We performed three independent trials and the data was shown as mean± SD.

### 2.10 Statistical analysis

We performed a statistical analysis using the SPSS 25.0 software. Correlations were analyzed using Spearman’s rank correlation coefficient. The student’s test was used to assess the difference between the two groups. We consider p < 0.05 as statistically significant.

## 3 Results

### 3.1 Biological characteristics of circMMD_007 in lung adenocarcinoma cells

**Figure 1.**
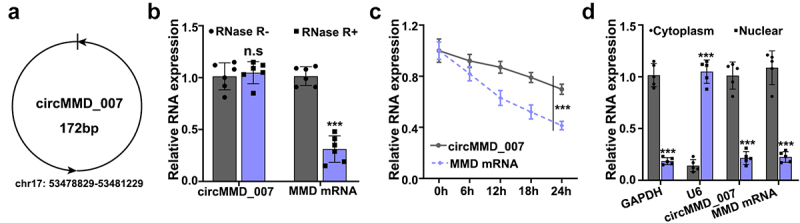
**The biological characteristics of hsa_circMMD_007 in lung adenocarcinoma cells**. A: The basic information of circMMD_007 was shown. B: The expression levels of circMMD_007 and UBAP2 mRNA were determined. C: UBAP2 mRNA and circMMD_007 expression were examined by qRT-PCR, after treating with Actinomycin. D: The location of circMMD_007 in A549 cells was determined.

According to circBank, the basic information of circMMD_007 is shown in Figure 1a. The expression levels of circMMD_007 and UBAP2 mRNA were determined by qRT-PCR. After treating with RNAase R, a statistically significant (p < 0.001) difference is observed, in Figure 1b. UBAP2 mRNA and circMMD_007 expressions were examined by qRT-PCR, after treating it with Actinomycin (Figure 1c). The location of circMMD_007 in A549 cells was determined, a statistically significant difference (p < 0.001) was observed (Figure 1d).

### 3.2 circMMD_007 expression was abnormally increased in lung adenocarcinoma

**Figure 2.**
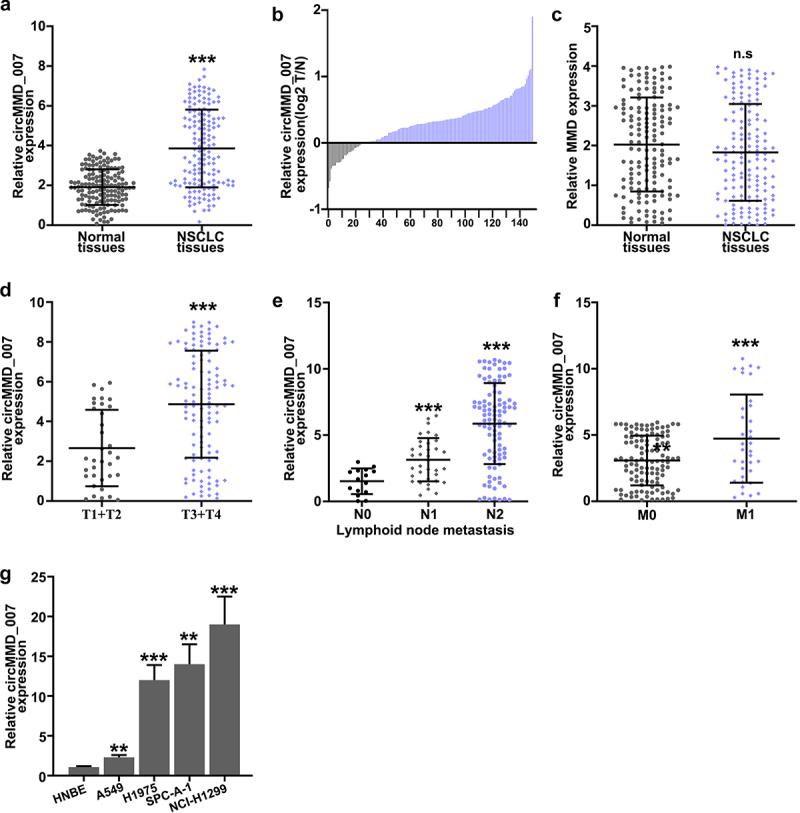
**Hsa_circMMD_007 was abnormally increased in lung adenocarcinoma** A, B: circMMD_007 was examined in lung adenocarcinoma and normal tissues by qRT-PCR. As compared with the normal tissues, hsa_cirMMD_007 expression was higher in NSCLC tissues. C: MMD was examined in lung adenocarcinoma and normal tissues. D: circMMD_007 expression was observed to be higher in (T3+ T4) lung adenocarcinoma tissues, as compared to (T1+ T2) lung adenocarcinoma tissues (p < 0.001). E: The expression was higher in N2 and N1, compared with N0. (p < 0.001). F: Expression was higher in the M1 stage, compared with M0 stage (p < 0.001). G: The expression of circMMD_007 was higher in NCI-H1299, NCI-H1395, A549, NCL-H460 cells, compared with HNBE cells (p < 0.01).

As compared with the normal tissues (p < 0.001), hsa_cirMMD_007 expression was higher in NSCLC tissues as shown in (Figures 2A, 2B). In Figure 2c, MMD was examined in LC specimens and normal tissues by qRT-PCR, the difference was found to be statistically insignificant (p > 0.05). Figure 2d illustrates that in T1 + T2 and T3 + T4 LC tissue, qRT-PCR was performed for circMMD_007 expression analysis. The expression of circMMD_007 was observed to be higher in (T3+ T4) LC tissues, as compared to (T1+ T2) LC tissues (p < 0.001). Figure 2e shows that in LC tissue including and excluding lymph node metastasis, qRT-PCR was performed for circMMD_007 expression analysis, the expression was higher in N2 and N1, compared with N0 (p < 0.001). Figure 2f shows that in the LC tissues of M0 and M1 stages, qRT-PCR was performed to analyze circMMD_007 expression. Expressions were higher in the M1 stage compared with the M0 stage (p < 0.001). Figure 2g shows that relative level of circMMD_007 was examined in HNBE, NCI-H1299, NCI-H1395, A549, NCL-H460 cells by qRT-PCR. circMMD_007 expression was higher in NCI-H1299, NCI-H1395, A549, NCL-H460 cells, compared with HNBE cells (p < 0.01).

### 3.3 In lung adenocarcinoma cells, circMMD_007 was specifically knocked out using shRNA

**Figure 3.**
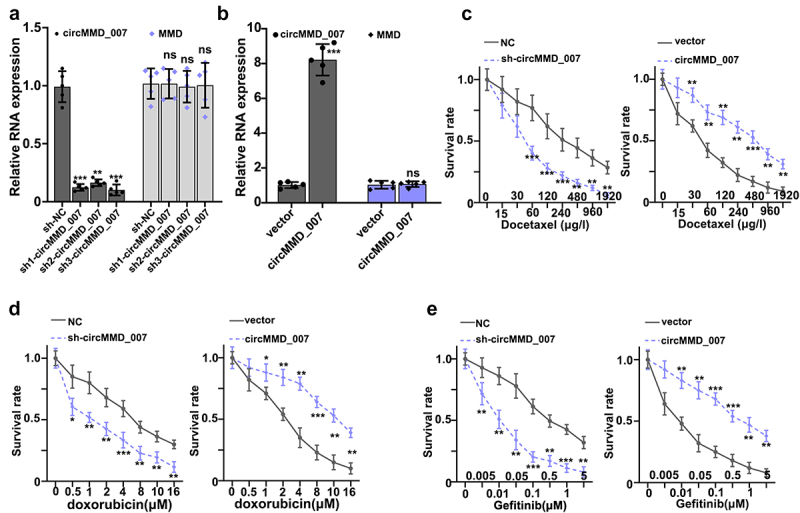
**In lung adenocarcinoma cells, hsa_circMMD_007 was specifically knocked out using shRNA**. A: Relative expression level of cirMMD_007 was lower in sh1-cirMMD_007, sh2-cirMMD_007, sh3-cirMMD_007 group (p < 0.01). Relative expression level of MMD was found to be statistically insignificant between the sh-NC, sh1-circMMD_007, sh2-circMMD_007, and sh3-circMMD_007 groups (p > 0.05). B: successful transfections of circMMD_007 overexpression and knockdown had been verified. C: When compared with the normal control group, lower survival rate in the sh-circMMD_007 group was observed, when treated with Docetaxel. In comparison with vector group (p < 0.01), higher survival rates were observed in circMMD_007 group. D: In comparison with normal control group, lower survival rate in the sh-circMMD_007 group was observed, when treated with Doxorubicin. In comparison with vector group (p < 0.01), higher survival rates were observed in circMMD_007 group. E: In comparison with normal control group, lower survival rate in the sh-circMMD_007 group was observed, when treated with Gefitinib. In comparison with vector group (p < 0.01), higher survival rates were observed in circMMD_007 group.

In Figure 3a, it was shown that when compared with the sh-NC group, the relative expression level of cirMMD_007 was lower in sh1-cirMMD_007, sh2-cirMMD_007, sh3-cirMMD_007 group (p < 0.01). The relative expression level of the MMD was found to be statistically insignificant between the sh-NC, sh1-circMMD_007, sh2-circMMD_007, and sh3-circMMD_007 groups (p > 0.05). In Figure 3b, successful transfections of circMMD_007 overexpression, and knockdown have been verified. Then, chemo resistance experiments on circMMD_007 knockdown and overexpression groups were performed. In Figure 3c, it was shown that in comparison with the normal control group, lower survival rate in the sh-circMMD_007 group was observed, when treated with Docetaxel (p < 0.01). In comparison, with vector group (p < 0.01), higher survival rates were observed in circMMD_007 group. In Figure 3d, it was shown that in comparison with the normal control group, lower survival rate in the sh-circMMD_007 group was observed, when treated with Doxorubicin (p < 0.01). In comparison, with vector group (p < 0.01), higher survival rates were observed in circMMD_007 group. In Figure 3e, it was shown that in comparison with the normal control group, lower survival rate in the sh-circMMD_007 group was observed, when treated with Gefitinib (p < 0.01). In comparison, with vector groups, higher survival rates were observed in circMMD_007 group (p < 0.01).

### 3.4 Knockout of hsa_circMMD_007 hindered the tumorigenesis of lung adenocarcinoma

**Figure 4.**
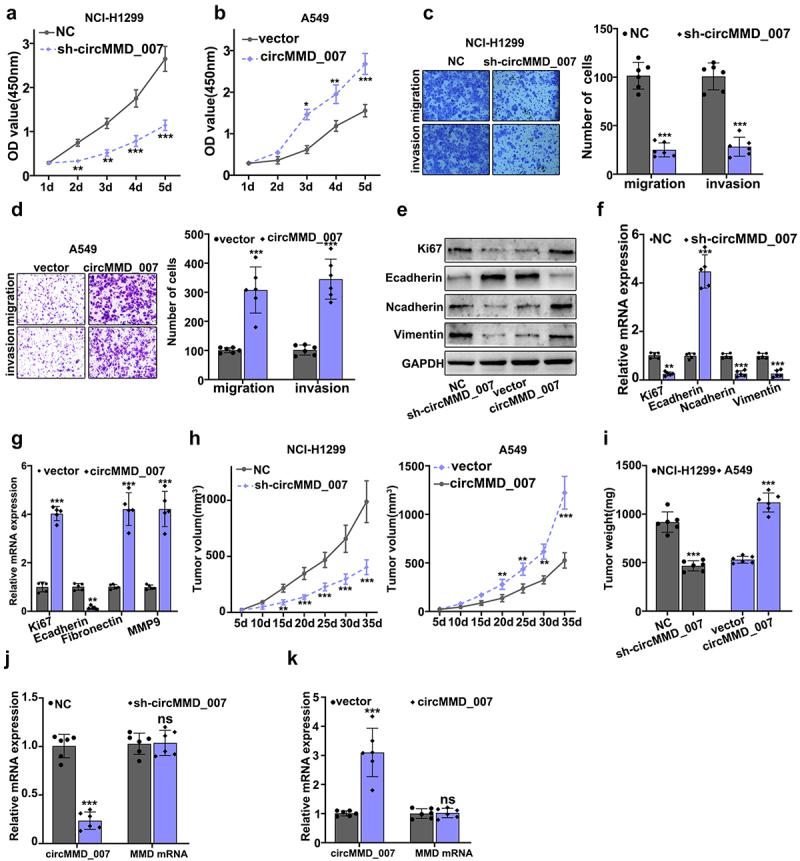
**Knockout of hsa_circMMD_007 hindered the tumorigenesis of lung adenocarcinoma in vivo and in vitro**. A-B: Considering NCI-H1299 cells, in comparison with the normal control (p < 0.001), the OD value was lower in sh-cirMMD_007. Considering A549 cells, when compared with the vector group, OD value was higher in cirMMD_007. C: When compared with the normal control group, the number of cells were lower in sh-cirMMD_007 group. D: When compared with the vector group, number of cells were higher in circMMD_007 group. E-G: Western blot and PCR were performed after regulating circMMD_007 expression. H: Considering NCI-H1299 cells, in comparison with the normal control (p < 0.01), the tumor volume was lower in sh-circMMD_007 group. Considering A549 cells, in comparison with the vector group (p < 0.01), the volume of the tumor was higher in circMMC_007 group. I: Considering NCI-H1299 cells, in comparison with the normal control (p < 0.01), the tumor weight was lower in sh-circMMD_007 group. Considering A549 cells, in comparison with the vector group (p < 0.01), the volume of the tumor was higher in circMMC_007 group. J: The expression of circMMD_007 was lower (p < 0.001), while the expression of UBAP2 mRNA exhibited no statistical difference. K: circMMD_007 expression was higher, while the expression of UBAP2 mRNA exhibited no statistical difference (p > 0.05).

Figures 4A-4B illustrate CCK8 experiment on circMMD_007 knockout and overexpression groups. Considering NCI-H1299 cells, in comparison with the normal control (p < 0.001), the OD value was lower in sh-cirMMD_007. Considering A549 cells, in comparison with the vector group (p < 0.05), OD value was higher in cirMMD_007 group. In Figure 4c, examination of migration and invasion ability was performed, after circMMD_007 knockdown in NCI-H1299 cells. While analyzing invasion and migration, it was observed that when compared with the normal control group, the number of cells was lower in sh-cirMMD_007 group (p < 0.001). Figure 4d illustrated migration and invasion was examined in circMMD_007 overexpression group in A549 cells. In comparison, with the vector group (p < 0.001), number of cells was higher in circMMD_007 group. Figure 4e-g shows that Western blot and PCR were performed after regulating circMMD_007 expression. Figure 4h illustrates xenograft tumor experiment on circMMD_007 knockdown and overexpression groups. Considering NCI-H1299 cells, in comparison with the normal control (p < 0.01), the tumor volume was lower in sh-circMMD_007 group. Considering A549 cells, in comparison with the vector group (p < 0.01), the volume of the tumor was higher in circMMC_007 group. In Figure 4i, illustrations regarding the tumor weight of circMMD_007 knockdown and overexpression groups are presented. Considering NCI-H1299 cells, in comparison with the normal control (p < 0.01), the tumor weight was lower in sh-circMMD_007 group. Considering A549 cells, in comparison with the vector group (p < 0.01), the volume of the tumor was higher in circMMC_007 group. In Figure 4j, illustration regarding the detection of circMMD_007 and UBAP2 mRNA in tumors in circMMD_007 knockdown group, is presented. The expression of circMMD_007 was lower (p < 0.001), while the expression of UBAP2 mRNA exhibited no statistical difference (p > 0.05). Figure 4k illustrates detection of circMMD_007 and UBAP2 mRNA in tumors in circMMD_007 overexpression group. The expression of circMMD_007 was higher (p < 0.001), while the expression of the UBAP2 mRNA exhibited no statistical difference (p > 0.05).

### 3.5 circMMD_007 negatively regulated the miR-197-3p expression

**Figure 5.**
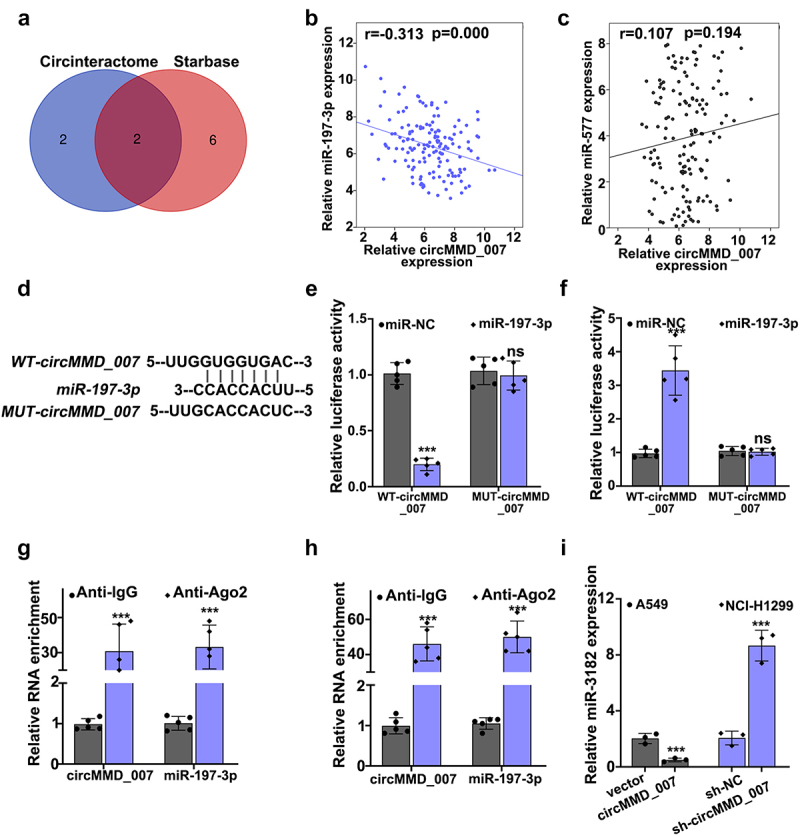
**Hsa_circMMD_007 negatively regulated the miR-197-3p expression**. A: Circinteractome and Starbase was used for the bioinformatics analysis of circMMD_007 and miR-197-3p. Venn diagram was drawn. B: Relative circMMD_007 expression was in negative interrelation with relative miR-197-3p expression (r = −0.313, p < 0.001). C-H: Binding between miR-197-3p and circMMD_007 was verified through RIP assay and dual luciferase reporter assay. I: In A549 cells, in comparison with vector group, relative miR-3182 expression was lower in circMMD_007 group. Considering NCI-H1299 cells, in comparison with the sh-NC group (p < 0.001), relative miR-3182 expression was higher in sh-circMMD-007 group.

Figure 5a depicts use of Circinteractome and Starbase for the bioinformatics analysis of circMMD_007 and miR-197-3p. A Venn diagram was drawn. Figure 5b shows the correlation between circMMD_007 and miR-197-3p in lung cancer tissue specimens. Relative circMMD_007 expression in negative correlation with relative miR-197-3p expression (r = −0.313, p < 0.001). Figures 5C-5 H illustrated that the binding between miR-197-3p and circMMD_007 was verified through dual-luciferase reporter assay and RIP assay. In A549 cell lines, Figure 5i shows that when compared with the vector group (p < 0.001), relative miR-3182 expression was lower in circMMD_007 group. Considering NCI-H1299 cells, in comparison with the sh-NC group (p < 0.001), relative miR-3182 expression was higher in sh-circMMD-007 group.

### 3.6 PTPN9 behaved as a molecular target of miR-197-3p

**Figure 6.**
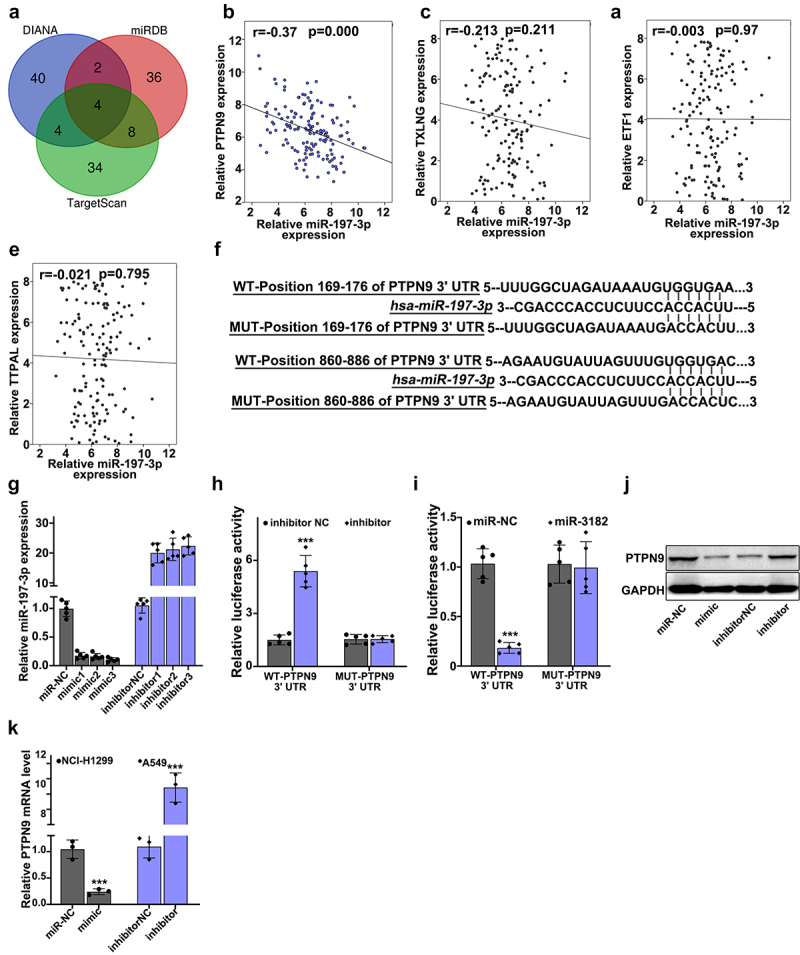
**PTPN9 behaved as a molecular target of miR-197-3p**. A: miR-197-3p target genes in 3 major databases (DIANA, miRDB and TargetScan) were searched, Venn diagram was drawn. B: a negative interrelation was shown between relative miR-197-3p expression and relative PTPN9 expression in lung cancer tissue specimens (r = −0.37, p < 0.001). C: a relationship was illustrated between the expressions of TXLNG and miR-197-3p in lung cancer tissue specimens (r = −0.213, p = 0.211). D: a relationship was illustrated between the expressions of ETF1 and miR-197-3p in lung cancer tissue specimens (r = −0.37, p = 0.97). E: a relationship was illustrated between the expressions of TTPAL and miR-197-3p in lung cancer tissue specimens (r = −0.37, p = 0.795). F: We applied dual luciferase reporter gene assay for verification of binding between PTPN9 and miR-197-3p. G:Best mimic and inhibitor sequence was selected. H-I: Target relationships were indicated by luciferase reporter experiment. J: For the evaluation of the effect of miR-197-3p up-regulation and down-regulation on PTPN9 expression, Western blot was performed. K: Compared with the miR-NC group, mimic group showed reduced PTPN9 mRNA expression. Compared with the inhibitor-NC group (p < 0.001), PTPN9 mRNA expression in inhibitor group was found to be higher.

Figure 6a illustrates that target genes of miR-197-3p in three major databases (DIANA, miRDB, and TargetScan) was searched, Venn diagram was drawn. In Figure 6b, a negative correlation was shown between relative miR-197-3p expression and the relative expression of PTPN9 in lung cancer tissue specimens (r = −0.37, p < 0.001). In Figure 6c, a relationship was illustrated between the expressions of TXLNG and miR-197-3p in lung cancer tissue specimens (r = −0.213, p = 0.211). In Figure 6d, a relationship was illustrated between the expressions of ETF1 and miR-197-3p in lung cancer tissue specimens (r = −0.37, p = 0.97). In Figure 6e, a relationship was illustrated between the expressions of TTPAL and miR-197-3p in lung cancer tissue specimens (r = −0.37, p = 0.795). Figure 6f illustrates the use of dual-luciferase reporter gene assay for the verification of binding between PTPN9 and miR-197-3p. In Figure 6g, the best mimic and inhibitor sequence was selected. In Figures 6H-6I, the target relationships were indicated by a luciferase reporter experiment. In Figure 6j, for the evaluation of the effect of miR-197-3p up-regulation and down-regulation on PTPN9 expression, Western blot was performed. In Figure 6k, relative PTPN9 mRNA expression was examined using qRT-PCR. Considering NCI-H1299 cells, when compared with the miR-NC group (p < 0.001), the mimic group showed reduced PTPN9 mRNA expression, considering A549 cells, when compared with the inhibitor-NC group (p < 0.001), PTPN9 mRNA expression in inhibitor group was found to be higher.

### 3.7 circMMD_007 had a carcinogenic effect in lung adenocarcinoma cells by governing the miR-197-3p/PTPN9 axis

**Figure 7.**
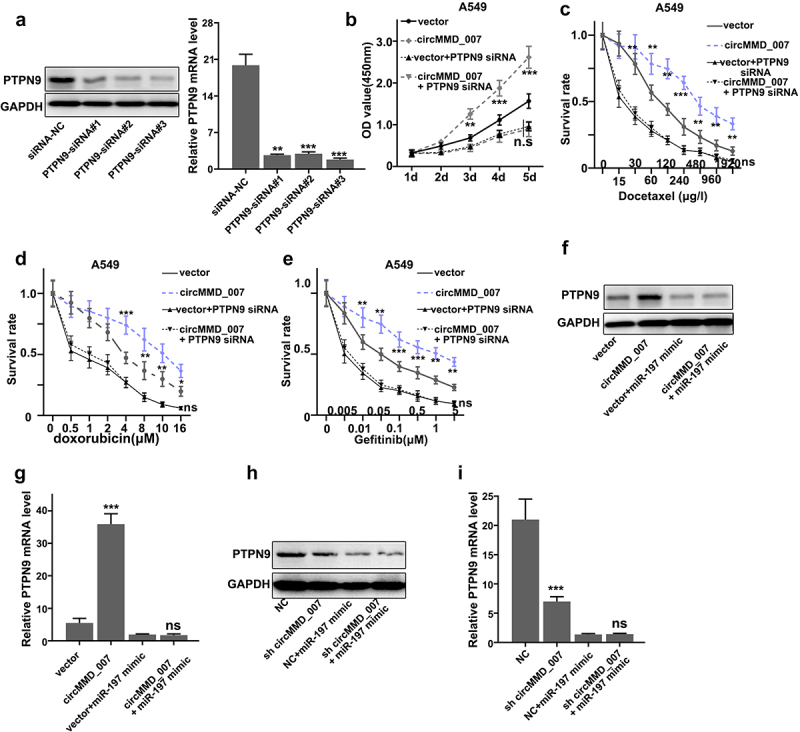
**Hsa_circMMD_007 had a carcinogenic effect in lung adenocarcinoma cells through governing the miR-197-3p/PTPN9 axis**. A: Interference was verified. B: After simultaneous transfection with PTPN9 siRNA in A549 cells, OD value in vector group (p < 0.001) was found to be lower in comparison with the OD value in circMMD_007 group. No difference between vector+PTPN9 siRNA group and circMMD_007+ PTPN9 siRNA group. C: When managed A549 cells with Docetaxel, the circMMD_007 group was higher in survival rate than vector group (p < 0.01), while no difference between vector+PTPN9 siRNA group and circMMD_007+ PTPN9 siRNA group. D: When managed A549 cells with Doxorubicin, the circMMD_007 group was higher in survival rate than vector group (p < 0.01), while no difference between vector+PTPN9 siRNA group and circMMD_007+ PTPN9 siRNA group. E: When managed A549 cells with Gefitinib, the circMMD_007 group was higher in survival rate than vector group (p < 0.01), while no difference between vector+PTPN9 siRNA group and circMMD_007+ PTPN9 siRNA group. F and H: Western blot was performed after transfecting miR-197-3p mimic. G: relative PTPN9 expression was detected using qRT-PCR, circMMD_007 group was higher in PTPN9 expression than the one found in the vector group (p < 0.001), no statistical difference was discovered between circMMD_007+ miR-197 mimic group and vector+miR-197 mimic group. I: Relative PTPN9 expression was detected using qRT-PCR, presence of PTPN9 expression was lower in sh-circMMD_007 group as compared to the normal control group (p < 0.001), no statistical difference was discovered between NC+miR-197 mimic group and sh circMMD_007+ miR-197 mimic group (p > 0.05).

Figure 7a shows that interference was verified by Western blot and qRT-PCR.

Figure 7b illustrates that after simultaneous transfection with PTPN9 siRNA in A549 cells, OD value in the vector group (p < 0.001) was found to be lower in comparison with the OD value in circMMD_007 group. The difference between vector+PTPN9 siRNA group and circMMD_007+ PTPN9 siRNA group was not statistically significant (p > 0.05). Figure 7c shows that when treated A549 cells with Docetaxel, the circMMD_007 group was higher in survival rate than the vector group (p < 0.01), while the difference between vector+PTPN9 siRNA group and circMMD_007+ PTPN9 siRNA group showed no statistical significance (p > 0.05). Figure 7d shows that when treated A549 cells with Doxorubicin, the circMMD_007 group was higher in survival rate than the vector group (p < 0.01), while the difference between vector+PTPN9 siRNA group and circMMD_007+ PTPN9 siRNA group was not significant (p > 0.05). Figure 7e shows that when treated A549 cells with Gefitinib, the circMMD_007 group was higher in survival rate than the vector group (p < 0.01), while difference between vector+PTPN9 siRNA group and circMMD_007+ PTPN9 siRNA group was not significant (p > 0.05). Figures 7F and 7 H show that Western blots were performed after transfecting miR-197-3p mimic. In Figure 7g, relative PTPN9 expression was detected using qRT-PCR, circMMD_007 group had higher PTPN9 expression than the one found in the vector group (p < 0.001), no statistical difference was discovered between circMMD_007+ miR-197 mimic group (p > 0.05) and vector+miR-197 mimic group. In Figure 7i, relative PTPN9 expression was detected using qRT-PCR, the presence of PTPN9 expression was lower in sh-circMMD_007 group as compared to the NC group (p < 0.001), no statistical difference was discovered between NC+miR-197 mimic group and sh circMMD_007+ miR-197 mimic group (p > 0.05)

## 4 Discussions

Circular RNAs consist of covalently attached continuous loops and are preserved across generations due to their inherent resistance toward RNA-degrading enzymes [15]. As compared to linear RNA, these non-coding types of circular RNA have better stability and are more resistant [16]. In this research, we discovered that the expression of circMMD_007 was abnormally increased in LC. Knockout of circMMD_007 hindered the tumorigenesis of LC in vivo and in vitro. circMMD_007 negatively regulated the miR-197-3p expression. PTPN9 was a molecular target of miR-197-3p. circMMD_007 had a carcinogenic effect in LC cells by regulating the miR-197-3p/PTPN9 axis.

Among the various microRNAs that have been investigated so far, miR-197-3p have been found to be dysregulated in a number of malignancies. For example, it is overexpressed in skin basal cell carcinoma and prevents keratinocyte proliferation and migration [17]. LIFR-AS1 could prevent the relocation and proliferation of breast cancer cells by binding with miR-197-3p [18]. To lower the chemo-sensitivity of cancerous cells in the lungs, long non-coding RNAs regulate miR-197-3p/p120 catenin axis [19]. In the case of liver cancer, miR-197-3p prevents cell invasion by acting as a suitable prognosis indicator [20].

PTPN9 is a constituent of the classical protein tyrosine phosphatases family, it is a protein phosphatase widely distributed in organisms [21]. Among the protein tyrosine phosphatases, it is considered distinctive because of its N-terminal Sec 14p homology domain, which helps in enzyme activation of the phosphatase domain of PTPN9 by binding phosphor-inositides [22]. It involves several cellular processes, such as intercellular junctions and signal transduction, and regulates various functions such as cell proliferation, migration, and differentiation. For instance, it stimulates fusion of secretory vesicles, controls endothelial cell function, facilitates insulin signaling in hepatocytes, and prevents cell growth in breast cancer [23]. It is obligatory for embryonic growth and development and expansion of erythroid cells. It has been also established that PTPN9 stimulated the dephosphorylation of EGFR and ErbB2 in breast tumor cells [24]. In recent times, PTPN9 was described to directly dephosphorylate STAT3 at the Tyr705 residue, which results in subsequent deactivation of STAT3 [25]. Inhibition of PTPN9 can improve migration and cell proliferation in gastric cancer and cervical cancer [21,26]. The literature also demonstrated that PTPN9 inhibited tumor progression in hepatocellular carcinoma, thus inhibits the progression of cancer, and mitigates the cell proliferation [27].

In the present study, we demonstrated that the expression of circMMD_007 was positively related to malignant degree of LC and distant lymph node metastasis. Knockdown of circMMD_007 could significantly suppress the tumor cell survival rate, but overexpression of circMMD_007 remarkably promoted cell survival rate after chemotherapy. In addition, the knockout of circMMD_007 markedly inhibited the tumorigenesis of LC, but the overexpression of circMMD_007 promoted tumor growth. The binding site between circMMD_007 and miR-197-3p was predicted, and the negative regulatory function was validated. In addition, the binding site between miR-197-3p and PTPN9 was predicted, and the negative regulatory function was validated. Finally, we demonstrated that circMMD_007 exert a carcinogenic effect on LC cells by governing the miR-197-3p/PTPN9 axis.

In further research, we tend to investigate the regulatory roles of miR-197-3p and PTPN9 in the LC tumor growth *in vivo*. Once the modulation functions of miR-197-3p and PTPN9 in LC *in vivo* are validated, we plan to explore the regulatory effect of circMMD_007/miR-197-3p/PTPN9 axis in LC using other kinds of animals. We finally want to apply circMMD_007/miR-197-3p/PTPN9 axis to the fields of LC diagnosis, prevention, and treatment.

## 5 Conclusions

In summary, this research demonstrated that circular RNA circMMD_007 could promote the oncogenic effects in the advancement of LC through miR-197-3p/PTPN9 axis. circMMD_007 might be as a new target for therapies of LC in the future.

## Data Availability

The datasets used and analyzed in the current study are available from the corresponding author in response to reasonable requests.
